# Supplementary material for: Effects of Alirocumab on Triglyceride Metabolism: A Fat-Tolerance Test and Nuclear Magnetic Resonance Spectroscopy Study
Source: Biomedicines. 2022 Jan 17;10(1):193. doi: 10.3390/biomedicines10010193 (PMC8774139; doi:10.3390/biomedicines10010193)
Supplement: Supplementary file 1 [file biomedicines-10-00193-s001.zip › biomedicines-1511486-supplementary.pdf]

Supplementary Materials

Abbreviations and Acronyms.

ANGPTL-3	Angiopoietin-like Protein 3
ANGPTL-4	Angiopoietin-like Protein 4
ApoB	Apolipoprotein B
ApoCII	Apolipoprotein C-II
ApoCIII	Apolipoprotein C-III
GPIHBP-1	Glycosylphosphatidylinositol-anchored High Density Lipoprotein-binding Protein-1
HDL	High Density Lipoprotein particles
LDL	Low Density Lipoprotein
Lp(a)	Lipoprotein a
PCSK9	Proprotein Convertase Subtilisin/Kexin type 9
VLDL	Very Low Density Lipoprotein

Table S1.

Title: Study population and alirocumab dosage.

Definition	Population (N)	Alirocumab 75 mg (%)	Alirocumab 150 mg (%)
Recruitment	24	19 (79.2)	5 (20.8)
Completion	19	16 (84.2)	3 (15.8)

Legend: Trial completion population (n=19) includes patients attending their baseline plus week 10 visit and injected alirocumab as planned. Three patients could not conduct their scheduled week 10 visit due to COVID-19 countermeasures. Two patients injected alirocumab only twice at baseline and at week 2 but not beyond.

Table S2

Title: Characteristics of all recruited trial participants.

Characteristic	All Recruited (N = 24)
Age - yr	66 (9)
Female sex - n (%)	9 (37.5)
Male sex - n (%)	15 (62.5)
Smoker[a] - n (%)	12 (50)
Current Smoker[b] - n (%)	5 (20.8)
Concomitant Diseases - n (%)	
Cardiovascular Disease	24 (100)
a. Coronary Heart Disease	23 (95.8)
Coronary Intervention or Surgery	19 (79.2)
Documentation of Coronary Stenosis[c]	4 (16.7)
b. Peripheral Artery Disease	3 (12.5)
c. Cerebral Artery Disease	8 (33.3)
Chronic Kidney Disease	5 (20.8)
Familial Hypercholesterolaemia[d]	4 (16.7)
Adiposity	4 (16.7)
Type-2 Diabetes Mellitus	4 (16.7)
Type-1 Diabetes Mellitus	0 (0)
Hypertension	19 (79.2)
Number of prior Cardiovascular Events[e] - n (%)	
Three	2 (8.3)
Two	6 (25)
One	12 (50)
Zero	4 (16.7)
Concomitant Lipid Medication – n (%)	
High-Intensity Statins[f]	5 (20.8)
Statins	7 (29.2)
Ezetimibe	15 (62.5)
Dietary Supplements[g]	6 (25)
Statin Intolerance[h]	19 (79.2)

Legend: Values are numbers (percentages) or means (standard deviations) for categorical and continuous variables, respectively. [a]Current or former smoker. [b]Documented as current smoker or no stop date documented. [c]Confirmed by cardiac computed tomography but without documentation of prior cardiovascular event (e.g., stroke, myocardial infarction, or percutaneous intervention). [d]According to medical records. [e]Documented as stent, balloon, coronary artery bypass graft, myocardial infarction, or percutaneous intervention, prior strokes/transient ischemic attacks. [f]Documented as ≥ 40 mg of atorvastatin or ≥ 20 mg of rosuvastatin. [g]Exclusively red yeast rice combination products (monacolin K). [h]Patients that did not receive high-intensity statins at baseline (includes partial or complete intolerance).

Figure S1

Title: Area under the curve (AUC) of triglycerides during fat-tolerance testing (t=0-4 hours) at baseline visit and after 10 weeks of alirocumab.

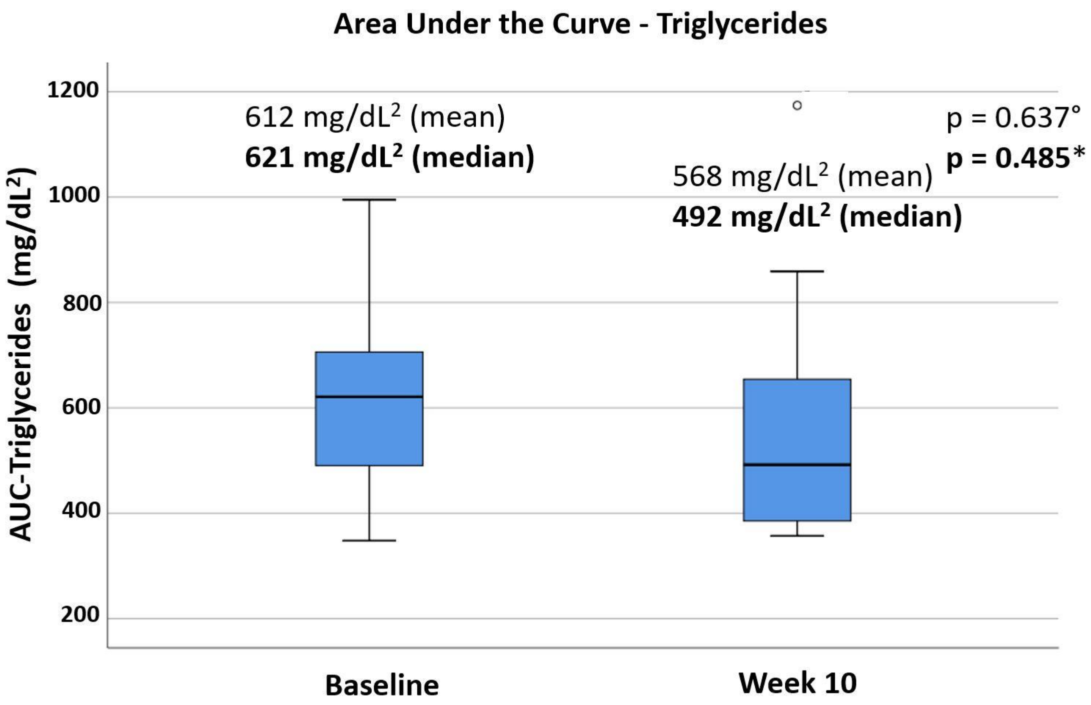

Legend: Among the trial-completion population, eleven patients had completed all measurements for AUC calculation (n = 11). Paired samples t-test with two-sided p-value. °Paired samples t-test; *Related samples Wilcoxon signed rank test. Calculations according to the trapezoid model.

Figure S2

Title: Distribution of VLDL particle size and changes by alirocumab treatment.

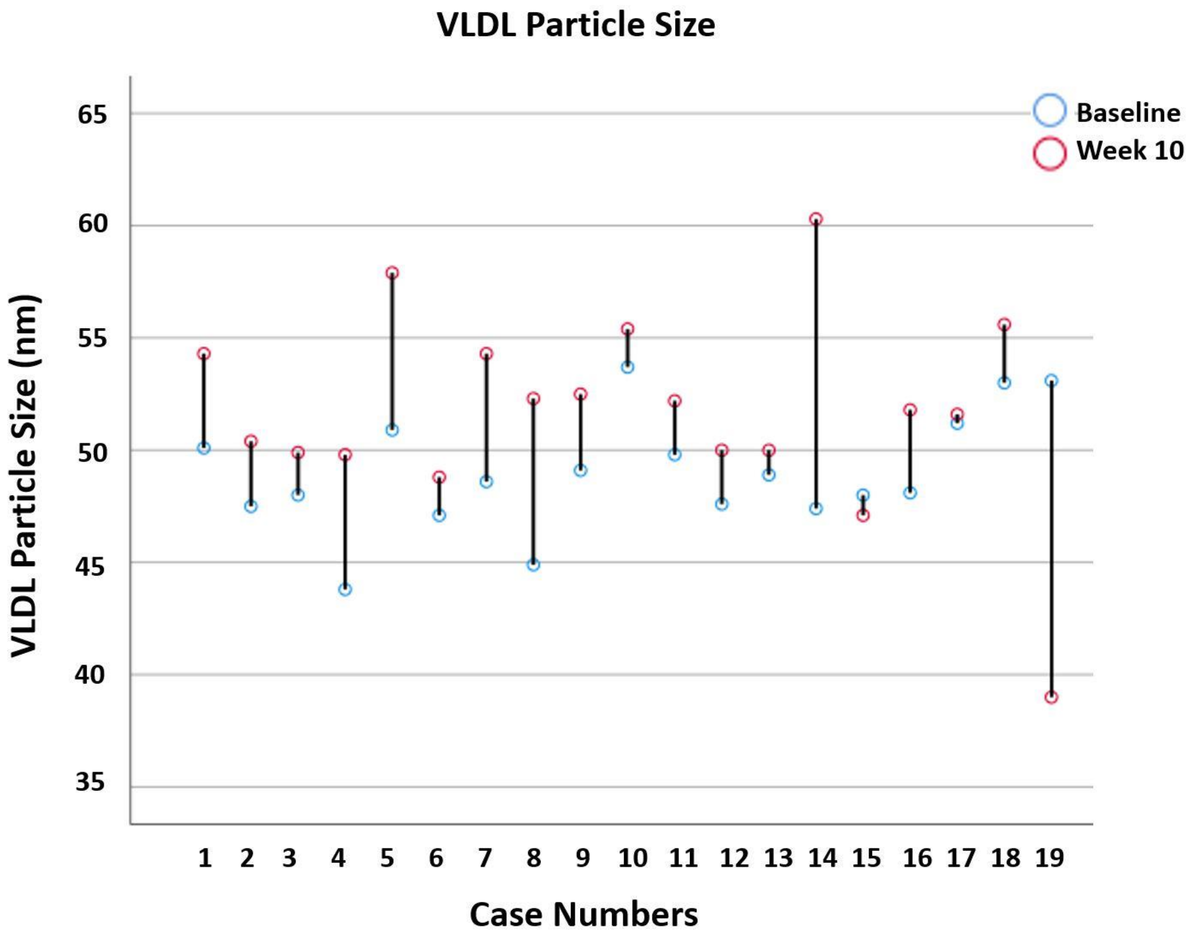

Legend: Shows nuclear magnetic resonance results of individual VLDL size and changes in response to alirocumab from baseline to week 10. Trial-completion population (n = 19). Case number 19 was a patient with mixed familial hyperlipidaemia and with baseline LDL-cholesterol of > 300 mg/dL, baseline small-dense LDL particles of 3500 nmol/L and with baseline triglyceride-rich large VLDL particles of 24 nmol/L.

Figure S3

Title: Distribution of small-dense LDL particles and changes by alirocumab treatment.

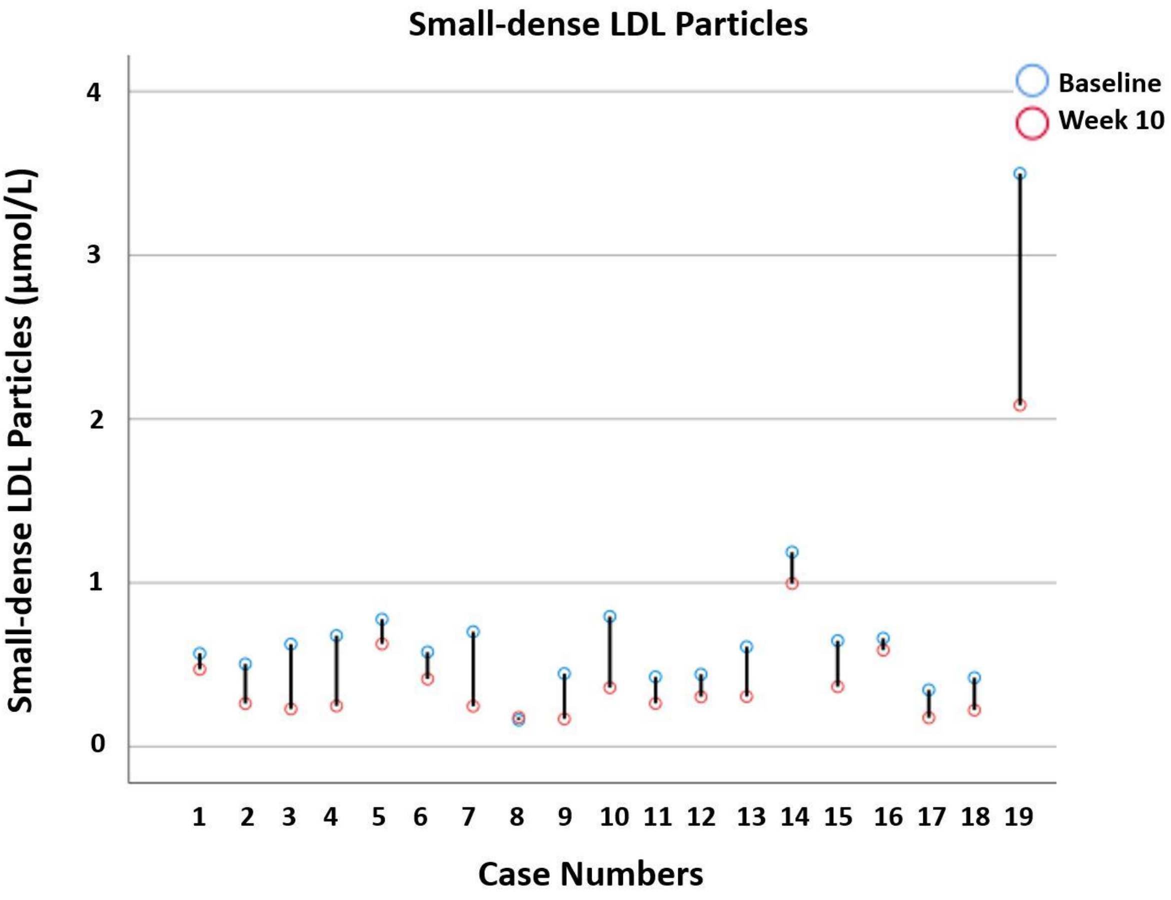

Legend: Shows nuclear magnetic resonance results of individual small-dense LDL particles and changes in response to alirocumab from baseline to week 10. Trial-completion population (n = 19). Case number 19 was a patient with mixed familial hyperlipidaemia and with baseline LDL-cholesterol of > 300 mg/dL, baseline small-dense LDL particles of 3500 nmol/L and with baseline triglyceride-rich large VLDL particles of 24 nmol/L.

Figure S4

Title: Plasma PCSK9 levels at baseline and after 10 weeks of alirocumab treatment.

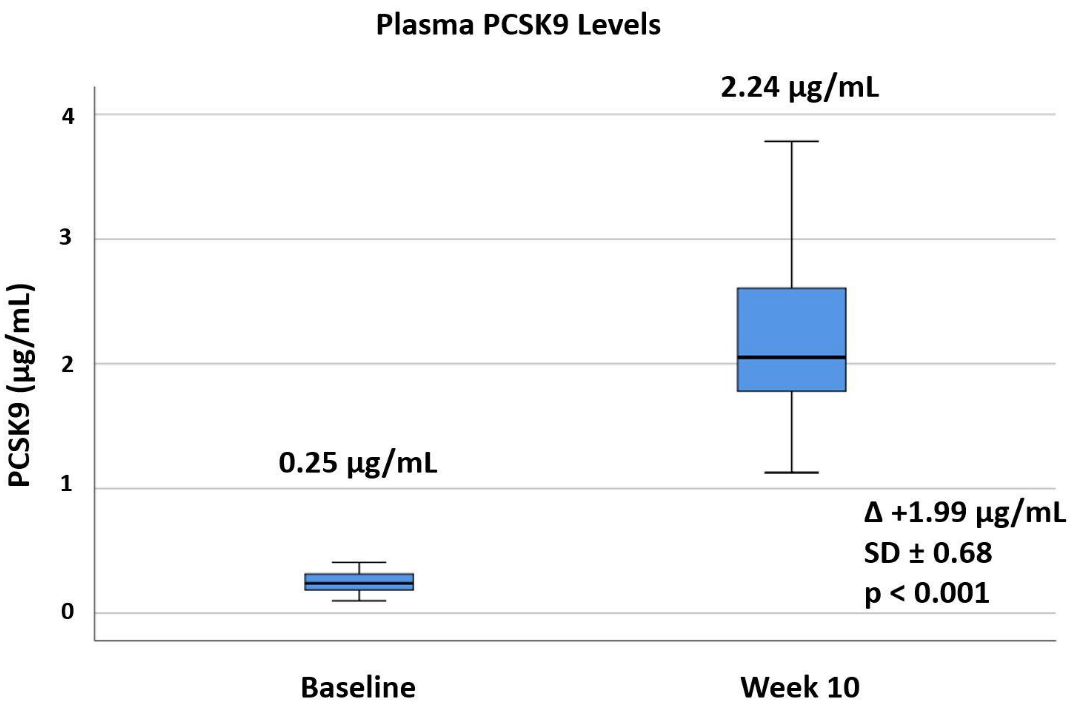

Legend: Figure shows trial-completion analysis (n = 19); Paired t-test with two-sided p-value; SD: Standard deviation.

Table S3

Title: LDL cholesterol and PCSK9 plasma levels per patient.

Method	Lipoprotein-Electrophoresis			Lipoprotein Analysis (Combined)			ELISA		
Patient-ID	LDL-C Baseline	LDL-C Week 10	LDL-C Change (%)	LDL-C Baseline	LDL-C Week 10	LDL-C Change (%)	PCSK9 Baseline	PCSK9 Week 10	X-Fold Increase
1	147	155	+5	145	176	+21	0.23	0.37	<2
2	90	48	-47	119	74	-38	0.31	2.53	8
3	128	41	-68	142	53	-63	0.41	3.70	9
4	81	27	-67	85	46	-46	0.31	1.83	6
5	242	34	-86	278	63	-77	0.19	2.86	15
6	161	67	-58	168	58	-65	0.36	2.84	8
7	154	78	-49	160	94	-41	0.17	1.77	11
8	193	57	-70	197	61	-69	0.21	2.22	10
9	125	60	-52	148	87	-41	0.22	2.59	12
10	59	27	-54	86	49	-43	0.31	1.13	4
11	126	46	-63	121	47	-61	0.10	1.32	13
12	90	11	-88	99	52	-47	0.14	1.65	11
13	116	76	-34	119	78	-34	0.18	1.45	8
14	160	92	-43	181	114	-37	0.24	2.03	8
15	206	91	-56	215	82	-62	0.23	1.98	9
16	165	68	-59	160	68	-58	0.25	1.79	7
17	173	140	-19	172	134	-22	0.27	2.63	10
18	100	35	-65	110	43	-61	0.14	2.05	15
19	79	80	+1	122	94	-23	0.16	0.25	<2
20	117	51	-56	132	64	-52	0.35	2.31	7
21	446	431	-3	319	433	+36	0.32	3.78	12

Legend: The table shows mean LDL-cholesterol (mg/dL) and mean PCSK9 (µg/mL) values per patient with week 10 assessments (n=21), as well as the relative impact of alirocumab treatment on these two parameters. Patients with the number 1 and 19 were non-adherent, thus did not inject alirocumab beyond week 2. Among participants with self-reported adherence, patient number 17 and 21 reflect low/non-response to alirocumab treatment but show high PCSK9 increase. Green: Lipoprotein-Electrophoresis; Blue: Lipoprotein analysis using the combined ultracentrifugation precipitation method; Purple: Enzyme-linked immunosorbent assay (ELISA). LDL-C: LDL-cholesterol.
